# Tracheotomy Successful in a Case of Pseudo-Membraneous Croup

**Published:** 1869-06

**Authors:** H. Wardner

**Affiliations:** Cairo, Ill.


### CHICAGO MEDICAL EXAMINER.

N. S. DAVIS, M.D., Editor.

VOL. X. JUNE, 1869. NO. 6.

### Original Contributions.

ARTICLE XXI.

### TRACHEOTOMY SUCCESSFUL IN A CASE OF PSEUDO-MEMBRANEOUS CROUP.

By H. WARDNER, M.D., of Cairo, Ill.

December 11th, 1868, was called to see Freddie, an infant son of Mr. G. E. Lounsbury, of Mound City, Ill. Learned that on the 9th he was attacked with croup, attended with spasms. On the 10th, all vocal sounds had ceased, and the struggle for life began in earnest. Every medicinal means was used that the present state of medical science could suggest, including emetics, sedatives, alteratives, local applications, lime vapor baths, etc., etc., but all in vain, except some temporary relief at times when the system was greatly relaxed by the treatment. Death seemed reaching for its victim.

It was so distressing to witness the child's struggles for breath, that tracheotomy was suggested to the parents, as a means of relief that would at least make an easier death, and would afford the only possible chance for life.

Assisted by Dr. N. R. Casey, I performed the operation, at half-past eleven o'clock, on the night of the 15th of December, being the sixth day of the disease. He was then just 13 months old, had a short, fat neck, which, with the small caliber of the soft and yielding trachea, caused the operation to be made slowly and with delicacy. I was prepared with two double canulas, the smallest of which was too large for the trachea. I then took out the inside tube, and succeeded in introducing it as a single canula. (I am thus particular, because the annoyances I met with may benefit others when in like circumstances.) At the first introduction of air through the tube, the child apparently fainted, and his breathing was intermitting for two or three minutes, when it became regular, and he dropped off into a quiet sleep. On the following day, he appeared much exhausted for a time, but soon began to revive and took some nourishment.

It was found very difficult to keep the single tube clear of mucous accumulations, consequently, a double tube was made as soon as possible by Mr. Tabor, an ingenious silversmith, of this city. On the fourth day after the operation, the tube was removed, and replaced by the newly prepared double canula, in the convex side of which was an aperture corresponding with the tracheal tube, for the purpose of admitting air through the larynx.

On the 8th day, the canula was removed, cleansed, and reädjusted. It was observed that the curve of the canula was too short, so much so that the lower extremity turned forward and pressed against the anterior wall of the trachea, evidently so as to produce an ulcer, as the discoloration of the silver showed that the sulphur of purulent matter must have come in contact with it at that point. This was partially remedied by placing a sort of cushion between the shield of the tube and the neck, thus keeping the tube from dropping in its full length.

On the 15th day, the tube was again removed, but had to be instantly replaced to prevent suffocation. With the coöperation of Dr. C. Gerieke, of St. Louis, I obtained a smaller canula, with a much longer curve, which was introduced on the 22d day. The patient was far more comfortable with this tube, as it did not press upon any very sensitive surface, nor impede deglutition.

At the end of the sixth week this was removed, but could not be dispensed with, and was at once replaced. Calomel was ordered, to be followed witn the daily administration of muriate of ammonia, and this was followed by tonics.

The patient being eight miles from my office, Dr. Hudson, Surgeon, U. S. N., on duty at Mound City, at my request, consented to watch the case daily. (In this connection, I wish to acknowledge the valuable services so courteously rendered by this intelligent officer during the remaining time of treatment.) Applications of nitrate of silver, 10 grains to the ounce of water, were made to the glottis, every three or four days, by Dr. H., who succeeded on several occasions in passing the probang into the larynx, each time being followed by marked improvement. Granular or polypoid growths were observed on the margin of the tracheal wound, which were touched with solid nitrate, through the aperture in the convexity of the tube. The growths were the frequent cause of severe paroxysms of coughing. At the end of 10 weeks, the tube was again removed, but had to be reinserted without delay.

The child's voice had partially returned, and he could breathe at times quite freely through the larynx. An article by Dr. Jacobi, in the *London Lancet*, led to the belief that these growths at the margin of the tracheal wound were the cause of suffocation on removal of the tube. They were, therefore, more freely cauterized; and, on the 7th of April, upon removing the tube, a small tumor came away with it. A marked improvement was observed this time; the patient was two or three minutes without the tube, and but little discoloration of face from want of air followed. The tube removed was replaced by another new one, made after a drawing by C. Degenhardt, of Chicago. This tube was just the thing, a much better fit than the last one. The patient had almost fully regained his voice, had learned to walk, and had cut seven teeth. I requested Dr. H. to remove the tube every four days, to mark the improvement, and leave it out entirely soon as it could be withdrawn with safety. On the 18th of April, 1869, just 128 days from the time of the operation, it was finally dispensed with. The opening closed within 72 hours. The *patient is well.*

I desire to acknowledge valuable suggestions in the treatment of this case from Dr. W. H. Byford, of Chicago, Dr. Paul F. Eve, of Nashville, and Dr. Abraham Jacobi, of New York City.

During the entire treatment, the child was kept in a large room, well ventilated, in which the thermometer was not allowed to vary five degrees day or night.

He was well fed on milk and such diet of a nourishing but digestible character as he would take.

The day before the operation, I gave him eight grains of calomel at one dose, feeling that I could afford to try anything in which there was the slightest possibility of relief; and I cannot help but think that this heroic dose stopped the disease from extending downwards into the lungs. I also believe that the polypoid or granular tumor on the margin of the tracheal wound was the cause of the tube being retained longer that it otherwise would have been had that not appeared.

The case also shows the necessity of having the canula made so as to fit the parts with the utmost precision compatible with circumstances. It also shows that life *may be* saved by the operation when all else fails.

